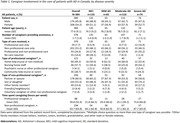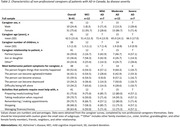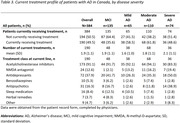# Treatment landscape and caregiver status for Alzheimer’s disease patients in Canada by disease severity: results from a real‐world survey

**DOI:** 10.1002/alz.095444

**Published:** 2025-01-09

**Authors:** Jennifer Glass, Chloe Walker, Luc Boulay, Brenda Botello Estrada, Sarah Cotton, Robert Laforce, Serge Gauthier, Jean‐Eric Tarride

**Affiliations:** ^1^ Eli Lilly Canada Inc., Toronto, ON Canada; ^2^ Adelphi Real World, Bollington, Cheshire United Kingdom; ^3^ Université Laval, Quebec, QC Canada; ^4^ McGill University, Montreal, QC Canada; ^5^ McMaster University, Hamilton, ON Canada

## Abstract

**Background:**

Caregiver burden associated with Alzheimer’s disease (AD) increases with AD severity, and current treatment options are limited. We aimed to describe the caregiver and treatment landscapes for patients with AD in Canada, split by disease severity.

**Method:**

Data were drawn from the Adelphi Real World AD Disease Specific Programme (DSP)™, a cross‐sectional survey of physicians in Canada, from March to October 2023. Physicians reported data on caregiver involvement in the care of patients with mild cognitive impairment (MCI)/AD and on patients’ current treatment profiles in patient record forms. Non‐professional caregivers of these patients were invited to complete a voluntary caregiver survey, which included caregivers’ characteristics. Patients were defined as having MCI or mild, moderate, or severe AD, according to current diagnostic label and physician‐reported disease severity. Analyses were descriptive.

**Result:**

Fifty physicians reported data on 384 patients with MCI/AD (mean age 77.6±9.1 years, 54.2% female). Data from 41 non‐professional caregivers was captured in the caregiver survey. The average number of caregivers per patient overall was 1.7 (1.0) and 2.1 (1.1) for patients with severe AD *(Table 1)*. Professional caregivers were primarily home help (49.2%). Most patients (77.8%) received non‐professional care. Partners cared for 60.5% of patients overall and 58.6% of patients with severe AD. Non‐professional caregivers spent 87.7±65.0 hours per week on average caring for patients with severe AD and 63.7±63.8 hours per week caring for patients overall. The symptom caregivers cited as causing them the most trouble was the patient forgetting recent events (52.6%) *(Table 2)*. Patients needed most help with preparing meals or cooking (75.6%). Physicians indicated that 49.5% of patients were receiving treatment for AD symptoms, primarily acetylcholinesterase inhibitors (91.1%; *Table 3*). Treatment was being received by 48.6% of patients with severe AD versus 61.8% of patients with moderate AD and 58.5% of patients with mild AD.

**Conclusion:**

The burden of care on the family of patients with AD in Canada is substantial. More caregivers and caregiver time were required on average for patients with severe AD than patients overall, and fewer patients with severe AD received treatment for AD.